# Analytical Sensitivity of Six SARS-CoV-2 Rapid Antigen Tests for Omicron versus Delta Variant

**DOI:** 10.3390/v14040654

**Published:** 2022-03-22

**Authors:** Jean-Louis Bayart, Jonathan Degosserie, Julien Favresse, Constant Gillot, Marie Didembourg, Happy Phanio Djokoto, Valérie Verbelen, Gatien Roussel, Céline Maschietto, François Mullier, Jean-Michel Dogné, Jonathan Douxfils

**Affiliations:** 1 Department of Laboratory Medicine, Clinique Saint-Pierre, 1340 Ottignies, Belgium; valerie.verbelen@cspo.be (V.V.); gatien.roussel@cspo.be (G.R.); 2 COVID-19 Federal Testing Platform and NGS Surveillance Consortium, CHU UCL Namur, 5530 Yvoir, Belgium; jonathan.degosserie@chuuclnamur.uclouvain.be (J.D.); celine.maschietto@chuuclnamur.uclouvain.be (C.M.); 3 Department of Laboratory Medicine, Université Catholique de Louvain, CHU UCL Namur, 5530 Yvoir, Belgium; francois.mullier@chuuclnamur.uclouvain.be; 4 Department of Laboratory Medicine, Clinique Saint-Luc, 5004 Namur, Belgium; j.favresse@labstluc.be; 5 Department of Pharmacy, Namur Research Institute for Life Sciences, University of Namur, 5000 Namur, Belgium; constant.gillot@unamur.be (C.G.); marie.didembourg@unamur.be (M.D.); phanio.djokoto@unamur.be (H.P.D.); jean-michel.dogne@unamur.be (J.-M.D.); jonathan.douxfils@unamur.be (J.D.); 6 Qualiblood S.A., 5000 Namur, Belgium

**Keywords:** SARS-CoV-2, variant, Omicron, Delta, antigen, RT-qPCR

## Abstract

Rapid antigen detection (RAD) tests are commonly used for the diagnosis of SARS-CoV-2 infections. However, with the continuous emergence of new variants of concern (VOC), presenting various mutations potentially affecting the nucleocapsid protein, the analytical performances of these assays should be frequently reevaluated. One hundred and twenty samples were selected and tested with both RT-qPCR and six commercial RAD tests that are commonly sold in Belgian pharmacies. Of these, direct whole-genome sequencing identified the strains present in 116 samples, of which 70 were Delta and 46 were Omicron (BA.1 and BA.1.1 sub-lineages, respectively). The sensitivity across a wide range of Ct values (13.5 to 35.7; median = 21.3) ranged from 70.0% to 92.9% for Delta strains and from 69.6% to 78.3% for Omicron strains. When taking swabs with a low viral load (Ct > 25, corresponding to <4.9 log_10_ copies/mL), only the Roche RAD test showed acceptable performances for the Delta strains (80.0%), while poor performances were observed for the other RAD tests (20.0% to 40.0%). All the tested devices had poor performances for the Omicron samples with a low viral load (0.0% to 23.1%). The poor performances observed with low viral loads, particularly for the Omicron strain, is an important limitation of RAD tests, which is not sufficiently highlighted in the instructions for use of these devices.

## 1. Introduction

Today, SARS-CoV-2 rapid antigen detection (RAD) tests are widely used in the ongoing COVID-19 pandemic [1]. Thanks to the short period of time between the test procedure and the availability of the result, they can lead to faster isolation of the patient and, therefore, limit virus spreading [2]. In Europe, the list of authorized RAD tests and their current fields of application can be found on the European Commission website, which stipulates the rules governing the authorization of these RAD tests on the European market [3]. While the analytical performances of these devices were initially reported according to these procedures, few of them have evaluated the impact of various variants of concern (VOC) on their claimed specifications [4,5]. Recently, the emergence of the Omicron variant was shortly followed by a release note from the Food Drug Administration (FDA), stating that RAD tests may have reduced sensitivity for the Omicron variant [6]. The European Commission adopted the same cautious approach, but stated, more precisely, that “[…] concerns have been raised about rapid antigen devices that are solely targeting the spike protein (thus not combined with the nucleocapsid protein) […]” [3]. This warning, along with reports of undetected Omicron-infected patients, led to controversy about the reliability of RAD tests in the Omicron wave [7]. Given the critical role that RAD tests play in early case detection, and their place in the testing strategy in many European countries, we studied and compared the performances of six RAD tests commonly sold in Belgium against the Delta and Omicron variants. 

## 2. Materials and Methods

Sixty positive specimens were randomly selected during the “Delta” wave (between 1 December and 5 December) and 60 other samples were selected during the Omicron wave (between 1 January and 6 January) at Clinique Saint-Pierre, Ottignies, Belgium. Nasopharyngeal swabs were collected using Vacuette Virus Stabilization tubes (Greiner Bio-One, Kremsmünster, Austria). Samples were thereafter heat inactivated by placing them in a drying oven for 15 min at 65 °C. RT-qPCR was performed using the Allplex^®^ 2019-CoV assay (Seegene, Arrow Diagnostics, Seoul, Korea). This method uses a volume of 300 µL and identifies SARS-CoV-2 RNA by targeting four viral genes (N, E and RdRP/S), thereby fulfilling internationally validated testing procedures [8]. Viral load was expressed as cycle threshold (Ct) and samples with Ct values < 37 for all three gene targets were considered as reactive for the present study. Ct values of the E gene were used as a proxy of the viral load. Thanks to validation with a quantified external reference standard, Ct values (x) can be transformed into viral loads (y: log_10_ copies/mL) according to the following formula: y = −0.2622x + 11.468 (R^2^ = 0.998). Therefore, a Ct value of 25 corresponds to 4.91 log_10_ RNA copies/mL (81.283 RNA copies/mL). According to this, samples can be classified into the following categories, according to their viral loads and, therefore, their potential infectivity:

- Weakly positive: <10^3^ RNA copies/mL (Ct > 32.29);
- Moderately positive: ≥10^3^ RNA copies/mL (Ct ≤ 32.29) to <10^5^ RNA copies/mL (Ct > 28.48);
- Strongly positive: ≥10^5^ RNA copies/mL (Ct ≤ 28.48) to <10^7^ RNA copies/mL (Ct > 17.04);
- Very strongly positive: ≥10^7^ RNA copies/mL (Ct ≤ 17.04).

Following RT-qPCR analysis, specimens were divided into several aliquots and frozen once at −20 °C until further analysis.

Among the 120 collected samples, 46 were confirmed by whole-genome sequencing (WGS) as being Omicron variant, while 70 were Delta variant. Four samples could not be identified due to insufficient material. Median age of the patients was 33 and 35 years old in the Delta and Omicron groups, respectively. Asymptomatic cases represented 24% (*n* = 17) and 37% (*n* = 16) of the Delta and Omicron groups, respectively. 

Whole-genome sequencing (WGS) was performed following the “midnight” method with the Oxford Nanopore Rapid Barcode library kit, as described elsewhere [9]. Following WGS, the sequences were aligned with complete SARS-CoV-2 genomes of different lineages through the NextClade online tool [10]. Subsequently, sequences were analyzed using the Phylogenetic Assignment of Named Global Outbreak Lineages (PANGOLIN) software. GISAID IDs of WGS are provided in Supplemental Material.

The following six RAD tests commonly sold in Belgian pharmacies were used: (i) the Clinitest^®^ Rapid COVID-19 Antigen test (Siemens Healthineers, Buizingen, Belgium), (ii) the New-gene COVID-19 Antigen Detection Kit (New-gene Bioengineering, Hangzhou, China), (iii) the Boson Rapid SARS-CoV-2 Antigen Test Card (Xiamen Boson Biotech Co., Xiamen, China), (iv) the Flowflex COVID-19 Antigen Home Test (Acon Laboratories, San Diego, CA, USA), (v) the Sejoy SARS-CoV-2 Antigen Rapid Test Cassette (Hangzhou Sejoy Electronics & Instrument Co., Hangzhou, China), and (vi) the Roche SARS-CoV-2 Rapid Antigen Test Nasal (Roche Diagnostics, Basel, Switzerland). All the tested RAD tests target the nucleocapsid protein and were intended for nasal self-sampling. The Clinitest RAD test also targets the S1, S1-RBD and S2 spike proteins, in addition to the nucleocapsid protein. 

Briefly, samples previously analyzed using RT-qPCR were unthawed and the appropriate number of droplets were dispensed on each device according to the recommendations of the manufacturers. Samples were identified as positive if both the control line and test line were present, as objectivated by two blinded and independent operators. The results of the six RAD tests were read after the reaction times recommended by the various manufacturers. 

Descriptive statistics were performed using GraphPad Prism^®^ software (version 9.0.1, San Diego, CA, USA) and MedCalc^®^ software (version 14.8.1, Ostend, Belgium). Sensitivity was defined as the proportion of samples qualified as positive by the RAD initially categorized as positive by RT-qPCR. A Student’s *t*-test was performed to assess statistical difference between cycle thresholds between RAD-positive and -negative groups. 

The study was in accordance with the Declaration of Helsinki and was approved by the ethical committee of the Clinique Saint-Pierre Ottignies (approval number: 2020-006149-21). Written informed consent was obtained for each patient. 

## 3. Results

The median Ct values were 20.83 and 21.37 in the Omicron and Delta groups, respectively (*p* = 0.37). Asymptomatic patients presented lower viral loads than symptomatic patients, both in the Delta group (median Ct; 31.1 vs. 19.8; *p* < 0.0001) and in the Omicron group (median Ct; 27.3 vs. 19.4; *p* < 0.0001). In the Omicron and Delta groups, 1 (2%) and 8 (11%) samples were weakly positive, 14 (30%) and 19 (27%) were moderately positive, 23 (50%) and 33 (47%) were strongly positive, and 8 (17%) and 10 (14%) were very strongly positive, respectively. 

Among the 70 samples identified as Delta, 67 could be analyzed through the PANGOLIN online tool, and were characterized as belonging to the following Delta sub-lineages: AY.109 (*n* = 1), AY.110 (*n* = 1), AY.112 (*n* = 1), AY.118 (*n* = 1), AY.122 (*n* = 6), AY. 123 (*n* = 1), AY. 127 (*n* = 8), AY.34.1 (*n* = 1), AY.4 (*n* = 11), AY. 4.2 (*n* = 3), AY.4.2.3 (*n* = 1), AY.42 (*n* = 1), AY.43 (*n* = 22), AY.44 (*n* = 1), AY.46 (*n* = 1), AY.46.6 (*n* = 1), AY.5 (*n* = 3), AY.9.2 (*n* = 1), AY.92 (*n* = 1) and B1.617.2 (*n* = 1), while the Omicron strains were composed of only the two following types: BA.1 (*n* = 40) and BA1.1 (*n* = 5). Three Delta strains and one Omicron strain could not be further characterized, due to technical limitations. 

**Figure 1:**
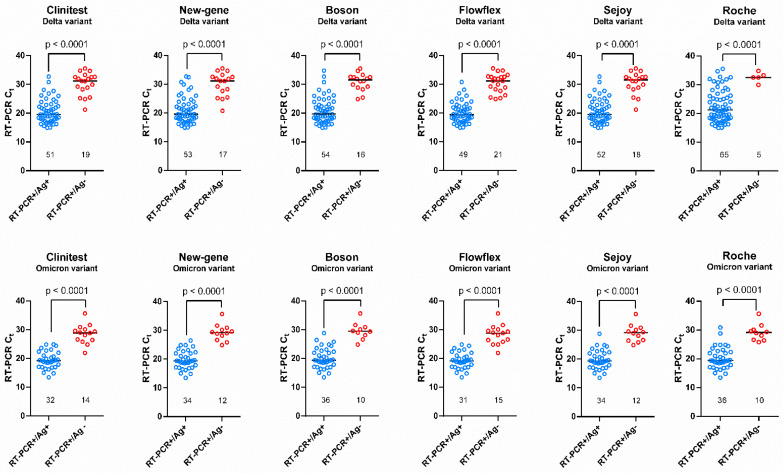
Graphical representation of positive and negative antigen results according to RT-qPCR Ct values. A significant difference in the Ct value was observed between the positive and negative tests for each RAD test.

Considering the whole cohort, the sensitivity of the RAD tests varied from 70.0% (Flowflex) to 77.1% (New-gene and Boson) for five out of six RAD tests, and was higher for the Roche device (92.9%) in the Delta group. The calculated sensitivities were similar across the RAD tests in the Omicron group, and varied from 69.6% (Clinitest) to 78.3% (Boson and Roche). The sensitivities among patients with higher viral loads (i.e., Ct ≤ 25) were also quite similar in the Omicron group, and varied between 91.2% (Flowflex) and 100% (Roche), versus 95.6% (Clinitest, New-gene and Sejoy) and 100% (Roche) in the Delta group. On the other hand, the sensitivities among samples with low viral loads (Ct > 25; *n* = 25 and *n* = 13 in the Delta and Omicron groups, respectively) were poor for both cohorts, except for the Roche RAD test, which exhibited good performances for the Delta strains (80.0% versus 20.0% to 40.0% for the other RAD tests). Two devices missed all the low viral load samples in the Omicron group (Clinitest and Flowflex), while the highest sensitivity (23.1%) was obtained with the Boson and Roche RAD tests, which were able to detect only 3 out of 13 samples (Table 1 and Figure 1). 

Taking only the asymptomatic cases into account, the calculated sensitivities were quite similar in the Omicron group, and varied from 31.3% (Clinitest and Flowflex) to 56.3% (Roche). In the Delta group, the Roche RAD test had the best performance (82.4%), while the sensitivity of the other devices was low, and ranged from 23.5% (New-gene and Flowflex) to 41.2% (Boson) (Table 1). 

The median Ct values between the positive and negative RAD results were systematically significative (*p* < 0.0001) in both groups (Figure 1).

## 4. Discussion

The rapid spread of the Omicron variant in November 2021 was quickly followed by several cases reporting false-negative RAD results [7,11]. Concomitantly, the FDA publicly warned that RAD tests may be less sensitive for the Omicron variant, which led to some concerns about using this strategy to screen the population [6]. It is still unclear whether these findings are due to mutations accumulated within the nucleocapsid of the virus, or whether they are due to variant-specific replication kinetics [12,13]. The latter explanation is further reinforced by several studies, which reported higher viral loads in saliva compared to paired nasal swabs [7,14]. In a high-risk occupational case cohort, Adamson et al. reported 30 cases where two RAD tests produced false-negative results on days 0 and 1, despite the fact that 28 out of these had potentially infectious viral loads (i.e., Ct < 29, as considered in the setting of this study). The median time between the first positive PCR and positive RAD test was 3 days, while the viral load in saliva peaked 1–2 days before the nose [7]. 

However, even if the Omicron viral load seems initially higher in saliva, RAD test performed with cheek or throat swabs alone seems to not be a good alternative, due to the significantly reduced sensitivity compared to a classical nasal swab [15,16]. Alternatively, nasal plus throat swabs increased the sensitivity from 64.5% to 88.7%, compared to the nasal swab only [15]. Moreover, some data emphasize the particular viral shedding of the Omicron variant, with increased sensitivity when RAD is performed several days following the first symptoms [7]. 

The sensitivities of the RAD tests for samples with Ct values ≤ 25 reported in this study are quite comparable to previous studies, and are in line with the expected performance of these assays, according to the clinical performance criteria for independent validation studies set up by the European HSC Technical Working Group on COVID-19 diagnostic tests [3,4,17,18,19]. Furthermore, in a systematic review and meta-analysis on the subject, Lee et al. reported a pooled sensitivity of 94%. However, most of the published studies could not address the VOC in their analyses, and whether distinct variants, with potential mutations affecting the nucleocapsid protein, could affect the sensitivity [20]. The reported sensitivities are largely variable across studies, and some manufacturers do not meet the current recommendations of the WHO, which request a sensitivity ≥ 80% and a specificity ≥ 97% [20]. 

The reported sensitivities of RAD in samples with Ct > 25 are frequently low. The pooled sensitivity of 38%, calculated by Lee et al., is in agreement with our values reported for the Delta variant, which vary from 20% to 40% for five out of the six evaluated RAD tests [20]. Only the Roche device demonstrated a satisfying performance, with a sensitivity of 80.0% in this population. Interestingly, in the samples containing Omicron strains, we found lower sensitivities, including for the Roche RAD test, ranging from 0% to 23.1%. These poor performances were also observed by Kanjilal et al. and Landaverde et al., who observed a sensitivity below 5% in detecting this category of samples (Ct > 25) with the widely used BinaxNOW^®^ RAD test. These studies were, however, performed on a limited number of samples, and did not compare several RAD tests [18,21]. 

Samples with a low viral load are commonly collected in asymptomatic patients. Five of the RAD tests evaluated in our study had low sensitivity (varying from 23.5% to 50%) in this population, both in the Omicron and Delta groups, while the Roche RAD test performed well for Delta strains (82.4%), but had quite comparable sensitivity for the Omicron samples (56.3%). These results are, therefore, globally in line with previous data [22,23]. These poor performances raise many questions about the testing strategy employed during the Omicron wave, where many asymptomatic patients were tested with RAD tests available in their local pharmacies, without confirmation with RT-qPCR in the case of a negative result. This may certainly have increased the spread of the virus among the population. 

Several studies have demonstrated the absence of variation in sensitivity for several RAD tests between major variants, including Alpha, Beta, Gamma, Delta and the ancestral SARS-CoV-2 strain [5,24,25]. However, due to the plethora of devices on the market, continuous surveillance on the analytical performances of these RAD devices is mandatory. Salcedo et al. reported a higher lowest limit of detection (LOD) for Delta (1000 PFU/mL) compared to Alpha, Gamma (10 PFU/mL) and Omicron (100 PFU/mL). While extensive data concerning Omicron are still limited, the initial experiments are reassuring, and suggest that the current mutations P13L, Del31-33, R203K and G204R, observed in the sequence of the N gene for the Omicron BA.1 and BA1.1 sub-lineages, do not affect the sensitivity of the commonly used RAD tests. On the other hand, Bekliz et al. found large heterogenicity between rapid diagnostic tests for detecting Omicron, with a sensitivity varying from 22.2% to 88.9%. Moreover, four out of the seven RAD tests evaluated showed significantly lower sensitivity in detecting Omicron when compared to Delta strains. These lower performances were observed both when assessed by infectious virus titers (PFU/mL) or by RNA copy numbers. While the design of this study was different from the present study, both observed reduced sensitivity of RAD in detecting Omicron in comparison to Delta strains [11]. 

Previous studies have identified specific mutations in the nucleocapsid, affecting the sensitivity of RAD. Jian et al. identified, in the Alpha variant, the T135I mutation as a cause of false-negative results with the Panbio COVID-19 RAD test [26]. Furthermore, as reported by Bourassa et al., some mutations can only affect specific devices; for example, the D399N nucleocapsid mutation only affected the Quidel Sofia SARS Antigen FIA test, but not the five other RAD tests, including the extensively used BinaxNOW COVID-19 Ag card [27]. Finally, in their local study in the region of Veneto (Italy), Del Vecchio et al. reported that, when analyzing the discordant results between RT-qPCR and RAD, a relevant fraction of the circulating variants contained A376T mutations coupled to M241I in the nucleocapsid sequence. Viruses harboring this sequence were over-represented in the antigen test-negative and PCR-positive samples, and their prevalence increased over time in this region, which had extensively used antigen tests [28]. This study underlines how mass utilization of RAD can create a selection pressure and enhance the spread of undetectable virus variants. In the present evaluation, WGS was not able to identify one or more specific mutations affecting the Omicron or Delta strains in the samples undetected by the various RAD tests. However, in light of our results, and those of Bekliz et al., at equivalent viral loads, we cannot firmly exclude the fact that RAD tests have lower sensitivity in detecting the Omicron variant compared to other VOC, especially the Delta variant. This could be due to a lower affinity of the monoclonal antibodies targeting the nucleocapsid in these devices. However, these antigenic targets are proprietary of the manufacturers, and it is, therefore, unclear whether certain binding affinities could be affected by the specific mutations concerning the Omicron variant. At the time of writing, BA.2 is now the main Omicron sub-lineage circulating in Belgium. While the present study included only BA.1 and BA.1.1 sub-lineages, BA.2 and BA.3 are characterized by an additional mutation (S413R), which currently has an unknown impact on RAD performances. 

The first limitation of this study is the sample modality, which was not fully identical to specific manufacturers’ recommendations, using a unique collection medium (Vacuette^®^; containing phosphate-buffered saline solution). This methodology was, however, applied for both strains and for all the tested RAD tests. Therefore, it is anticipated that the comparison of their analytical performances remains valid. The second main limitation of this study is the use of heat-inactivated samples that underwent one freeze—thaw cycle prior to the assessments by RAD. However, as described by Loveday et al., heat inactivation eliminates infectivity, but leaves virions mostly intact, while modest reduced antigenicity can be observed, potentially impacting the measured sensitivity. 

## 5. Conclusions

In the present study, all the devices presented good performances for detecting both Delta and Omicron variants in patients with high viral loads (Ct ≤ 25), with sensitivities varying from 91.2% to 97.8%, in line with the expectations of the European HSC Technical Working Group on COVID-19 diagnostic tests. RAD showed poor performance for patients with low viral loads (Ct > 25) and in asymptomatic patients, except for the Roche device, which showed higher performances in the Delta group. The sensitivity among Omicron strains was poor and lower than for Delta, including for the Roche RAD test, and some devices did not detect any Omicron samples presenting Ct > 25. The current testing strategy of not confirming negative RAD testing by RT-qPCR in asymptomatic cases certainly increased the spread of the pandemic these last months. However, this fact does not invalidate the benefits of the testing strategy in the current pandemic, in terms of accessibility, ease and rapidity of use. However, the limited performances observed for RAD suggests that symptomatic and high-risk contact cases should have a RT-qPCR confirmation in the case of a negative result. Conversely, situations where the likelihood of infection is lower, such as asymptomatic cases or tests performed for social events, should not require confirmation, given the lower risk of infection and the short turnaround time required in the latter setting. Finally, the apparent lower sensitivity of these devices for the Omicron variant raises the question of whether the current commercialized RAD should be reevaluated, or even further readapted to the current and future genomic changes observed in the virus.

## Figures and Tables

**Table 1:** Comparison of the sensitivity of 5 RAD tests between the Delta and Omicron variants. Calculated sensitivities, along with 95% confidence intervals (in brackets), are given.

Population	Variant	Clinitest	New-Gene	Boson	Flowflex	Sejoy	Roche
**Total cohort**	**Delta**	72.9%	75.7%	77.1%	70.0%	74.3%	92.9%
	**(*n* = 70)**	(60.9–82.8)	(64.0–85.2)	(65.6–86.3)	(57.9–80.4)	(62.4–84.0)	(84.1–97.6)
	**Omicron**	69.6%	73.9%	78.3%	67.4%	73.9%	78.3%
	**(*n* = 46)**	(54.3–82.3)	(58.9–85.7)	(63.6–89.1)	(52.0–80.5)	(58.9–85.7)	(63.6–89.1)
**Ct ≤ 25**	**Delta**	95.6%	95.6%	97.8%	97.8%	95.6%	100%
	**(*n* = 45)**	(84.9–99.5)	(84.9–99.5)	(88.2–99.9)	(88.2–99.9)	(84.9–99.5)	(92.1–100)
	**Omicron**	94.1%	97.1%	97.1%	91.2%	97.1%	100%
	**(*n* = 33)**	(80.3–99.3)	(84.7–99.9)	(84.7–99.9)	(76.3–98.1)	(84.7–99.9)	(89.4–100)
**Ct > 25**	**Delta**	32.0%	40.0%	40.0%	20.0%	36.0%	80.0%
	**(*n* = 25)**	(15.0–53.5)	(21.1–61.3)	(21.1–61.3)	(6.8–40.7)	(18.0–57.5)	(59.3–93.2)
	**Omicron**	0.0%	7.7%	23.1%	0.0%	7.7%	23.1%
	**(*n* = 13)**	(0.0–24.7)	(0.2–36.0)	(5.0–5.38)	(0.0–24.7)	(0.2–36.0)	(5.0–53.8)
**Asymptomatic**	**Delta**	35.3%	23.5%	41.2%	23.5%	35.3%	82.4%
	**(*n* = 17)**	(14.2–61.7)	(6.8–49.9)	(18.4–67-1)	(6.8–49.9)	(14.2–61.7)	(56.6–96.2)
	**Omicron**	31.3%	37.5%	50.0%	31.3%	43.8%	56.3%
	**(*n* = 16)**	(11.0–58.7)	(15.2–64.6)	(24.7–75.4)	(11.0–58.7)	(19.8–70.1)	(29.9–80.3)
**Symptomatic**	**Delta**	84.9%	92.5%	88.7%	84.9%	86.8%	96.2%
	**(*n* = 53)**	(72.4–93.3)	(81.8–97.9)	(77.0–95.7)	(72.4–93.3)	(76.7–94.5)	(87.0–99.5)
	**Omicron**	90.0%	93.3%	93.3%	86.7%	90.0%	90.0%
	**(*n* = 30)**	(73.5–97.9)	(77.9–99.2)	(77.9–99.2)	(69.3–96.2)	(73.5–97.9)	(73.5–97.9)

## Data Availability

The data presented in this study are available on request from the corresponding author.

## Supplementary Materials

The following are available online at https://www.mdpi.com/article/10.3390/v14040654/s1, GISAID’s ID of the strains included in the RAD comparison*.
