# Supplementary material for: Analytical Sensitivity of Six SARS-CoV-2 Rapid Antigen Tests for Omicron versus Delta Variant
Source: Viruses. 2022 Mar 22;14(4):654. doi: 10.3390/v14040654 (PMC9031584; doi:10.3390/v14040654)
Supplement: Supplementary file 1 [file viruses-14-00654-s001.zip › viruses-1633151-supplementary.pdf]

GISAID's ID of the strains included in the RAD comparison* :

EPI_ISL_10945573
EPI_ISL_10942641
EPI_ISL_10942640
EPI_ISL_10942639
EPI_ISL_10942645
EPI_ISL_10942741
EPI_ISL_10945571
EPI_ISL_10942647
EPI_ISL_10942651
EPI_ISL_10942650
EPI_ISL_10945572
EPI_ISL_10942644
EPI_ISL_10942675
EPI_ISL_10942717
EPI_ISL_10942673
EPI_ISL_10942716
EPI_ISL_10942652
EPI_ISL_10942670
EPI_ISL_10942642
EPI_ISL_10942668
EPI_ISL_10942711
EPI_ISL_10942731
EPI_ISL_10942713
EPI_ISL_10942689
EPI_ISL_10942666
EPI_ISL_10942708
EPI_ISL_10942677
EPI_ISL_10942728
EPI_ISL_10942665
EPI_ISL_10945569
EPI_ISL_10942740
EPI_ISL_10942686
EPI_ISL_10942705
EPI_ISL_10942648
EPI_ISL_10942704
EPI_ISL_10942643
EPI_ISL_10942684
EPI_ISL_10942746
EPI_ISL_10942744
EPI_ISL_10942703

EPI_ISL_10942743
EPI_ISL_10942702
EPI_ISL_10942701
EPI_ISL_10942682
EPI_ISL_10942680
EPI_ISL_10942742
EPI_ISL_10942700
EPI_ISL_10942678
EPI_ISL_10942699
EPI_ISL_10942738
EPI_ISL_10942698
EPI_ISL_10942739
EPI_ISL_10942646
EPI_ISL_10942697
EPI_ISL_10942696
EPI_ISL_10942695
EPI_ISL_10942737
EPI_ISL_10942694
EPI_ISL_10942736
EPI_ISL_10942693
EPI_ISL_10942692
EPI_ISL_10942735
EPI_ISL_10942691
EPI_ISL_10942734
EPI_ISL_10942649
EPI_ISL_10942733
EPI_ISL_10945570
EPI_ISL_10942732
EPI_ISL_10942745
EPI_ISL_10942729
EPI_ISL_10942730
EPI_ISL_10942688
EPI_ISL_10942727
EPI_ISL_10942657
EPI_ISL_10942687
EPI_ISL_10942726
EPI_ISL_10942685
EPI_ISL_10942683
EPI_ISL_10942681
EPI_ISL_10942725
EPI_ISL_10942724
EPI_ISL_10942723
EPI_ISL_10942722

EPI_ISL_10942679
EPI_ISL_10942721
EPI_ISL_10942672
EPI_ISL_10942720
EPI_ISL_10942676
EPI_ISL_10942719
EPI_ISL_10942674
EPI_ISL_10942667
EPI_ISL_10942669
EPI_ISL_10942671
EPI_ISL_10942690
EPI_ISL_10942663
EPI_ISL_10942718
EPI_ISL_10942661
EPI_ISL_10942715
EPI_ISL_10942664
EPI_ISL_10942662
EPI_ISL_10942660
EPI_ISL_10942714
EPI_ISL_10942712
EPI_ISL_10942656
EPI_ISL_10942658
EPI_ISL_10942710
EPI_ISL_10942655
EPI_ISL_10942654
EPI_ISL_10942709
EPI_ISL_10942653
EPI_ISL_10942707
EPI_ISL_10942706
EPI_ISL_10942659

*2 samples had too low quality to be accepted.
